# Metformin inhibits high glucose-induced smooth muscle cell proliferation and migration

**DOI:** 10.18632/aging.102955

**Published:** 2020-03-24

**Authors:** Dong-Ming Zhou, Feng Ran, Hai-Zhen Ni, Li-Li Sun, Lun Xiao, Xiao-Qiang Li, Wen-Dong Li

**Affiliations:** 1 Department of Hematology, The Affiliated Drum Tower Hospital, Nanjing University Medical School, Jiangsu, China; 2 Department of Vascular Surgery, The Affiliated Drum Tower Hospital, Nanjing University Medical School, Jiangsu, China; 3 Department of Vascular Surgery, The Second Affiliated Hospital of Soochow University, Jiangsu, China; 4 Department of Vascular Surgery, The First Affiliated Hospital of Wenzhou Medical University, Zhejiang, China

**Keywords:** metformin, smooth muscle cells, HMGB1, autophagy, miR-142-3p

## Abstract

We investigated the protective effects and mechanism of action of metformin on high glucose-induced smooth muscle cell proliferation and migration. Vascular smooth muscle cells (VSMCs) were subjected to a series of concentrations (0-10 mM) of metformin. CCK-8, wound healing, and transwell assays were performed. Correlations between metformin concentration and high-mobility group box 1 (HMGB1) and miR-142-3p levels were assessed. In addition, miR-142-3p mimic and siRNA were used to investigate VSMC migration in the presence or absence of metformin. In the high-glucose condition, metformin decreased cell growth and inhibited cell migration. HMGB1 gene expression correlated negatively with metformin concentration, whereas miR-142-3p expression correlated positively with metformin concentration. In addition, mimic-induced miR-142-3p elevation resulted in decreased HMGB1 and LC3II levels and elevated p62 levels in the high-glucose condition, whereas miR-142-3p knockdown had the reverse effects, and metformin abolished those effects. Metformin inhibits high glucose–induced VSMC hyperproliferation and increased migration by inducing miR-142-3p-mediated inhibition of HMGB1 expression via the HMGB1-autophagy related pathway.

## INTRODUCTION

Arteriosclerosis obliterans (ASO) is a major cause of death and disability, particularly in patients with diabetes mellitus (DM) [1, 2]. This is because the inflammation underlying atherosclerosis exacerbated by hyperglycemic states [3]. High-mobility group box 1 (HMGB1), is a crucial inflammatory factor in atherosclerosis [4–6], and a danger signal for vascular disease [7]. Abundant HMGB1 within carotid and coronary atherosclerotic plaques [8] contributes to the abnormal proliferation and migration of vascular smooth muscle cells (VSMCs) [9, 10]. This makes HMGB1 inhibition potentially effective theraputic approach to atherosclerosis.

MicroRNAs affect gene expression, and their dysregulation increases inflammation and leads to atherosclerosis [11–13]. Studies have shown that many microRNAs, including miR- 504, -200, -138 and -210, regulate the proliferation and migration of VSMCs [3]. Furthermore, studies have determined that some microRNAs inhibit the expression of HMGB1 [14–16]. Thus inhibiting HMGB1 expression via microRNAs in VSMCs may be a therapeutic approach to reduce atherothrombotic events.

Metformin, which is used to treat type 2 DM, may suppress diabetes-accelerated atherosclerosis [17, 18] via AMPK-mediated inhibition of VSMC proliferation and migration [19]. Metformin’s promotion of microRNA expression and anti-inflammatory effects also contribute to this process [19–21]. In prior studies, metformin was found to inhibit the expression and release of HMGB1 [22, 23]. We therefore speculated that metformin may inhibit high glucose–induced VSMC proliferation and migration via microRNA–mediated inhibition of HMGB1 expression. To test that idea, VSMCs were isolated from the rat aorta and characterized by fluorescence microscopy and subjected to a high-glucose environment and a series metformin concentrations (0-10 mM). Our findings suggest metformin inhibits glucose-induced VSMC hyperproliferation and migration by inhibiting HMGB1 expression via the HMGB1-autophagy related pathway

## RESULTS

### Metformin inhibited high glucose–induced VSMC hyperplasia in a dose-dependent manner

**Figure 1:**
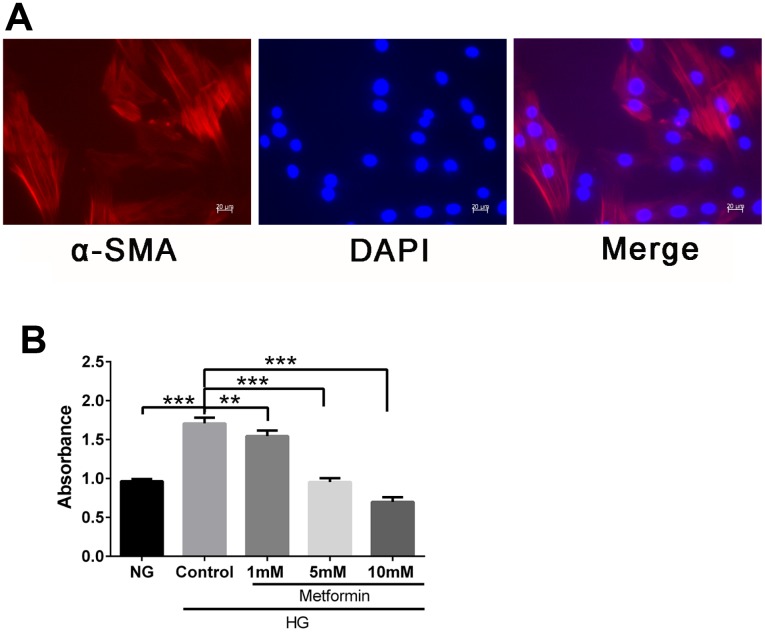
**Metformin inhibited high glucose–induced vascular smooth muscle cell (VSMC) hyperplasia in a dose-dependent manner.** (**A**) VSMC characterization by fluorescence microscopy. Expression of smooth muscle cell marker α-SMA was confirmed by red fluorescence. (**B**) CCK-8 evaluation of the effects of metformin on VSMC proliferation. Significant inhibition of high glucose–induced cell proliferation was found at metformin concentrations of 1, 5, and 10 mM. **p < 0.01 and ***p < 0.001 for between-group comparisons.

To evaluate the effects of metformin on VSMCs in a high-glucose condition, we first isolated and characterized the VSMC and confirmed the presence of the VSMC marker α-SMA in these cells (Figure 1A). We then subjected the cells to a high-glucose environment and examined the cell proliferation. Finally, metformin was added, and the results showed that metformin at concentrations of 1, 5, and 10 mM significantly inhibited high glucose–induced VSMC hyperproliferation (Figure 1B).

### Metformin inhibited high glucose-induced VSMC migration via the HMGB1-autophagy related pathway

**Figure 2:**
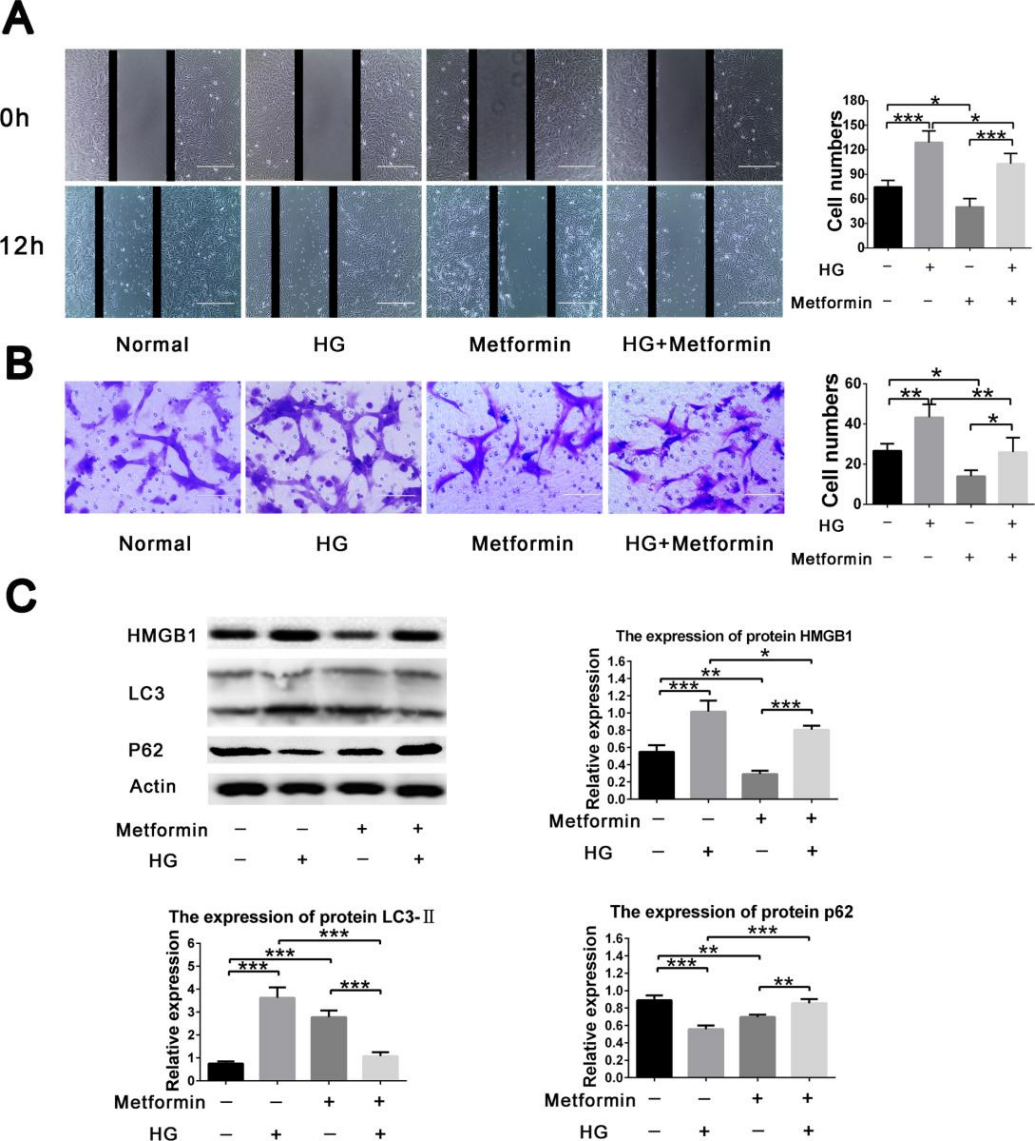
**Metformin inhibited high glucose–induced vascular smooth muscle cell (VSMC) migration via HMGB1-autophagy related pathway.** (**A**) Metformin inhibited high glucose–induced VSMC migration in a two dimensional scratch assay. Significant inhibition of cell migration was found when metformin was added in the high-glucose condition; in addition, metformin resulted in decreased cell migration in the normal glucose condition. (**B**) Metformin inhibited high glucose–induced VSMC migration in a three dimensional transwell assay. Significant inhibition of cell migration was found when metformin was added in the high-glucose condition; in addition, metformin resulted in decreased cell migration in the normal glucose condition. (**C**) The HMGB1-autophagy related pathway was involved in the effects of metformin on high glucose–induced cell migration. Metformin resulted in decreased HMGB1 and LC3II levels and increased the p62 level in the high-glucose condition. In addition, metformin resulted in decreased HMGB1 and p62 levels and increased LC3II level in the normal glucose condition. *p < 0.05, **p < 0.01 and ***p < 0.001 for between-group comparison.

To further evaluate the effects of metformin on high glucose–induced VSMC behavior, a two dimensional scratch assay and three dimensional transwell assay were performed. The results showed significant inhibition of high glucose–induced cell migration when metformin was added to the assays. Metformin also resulted in decreased cell migration under normal glucose condition (Figure 2A, 2B). In addition, we also evaluated the expression of HMGB1-autophagy related pathway molecules and found that metformin treatment resulted in inhibition of the elevated HMGB1 and LC3II levels and the decreased p62 level found in the high-glucose condition. Similarly, the addition of metformin resulted in decreased HMGB1 and LC3II levels and increased p62 level in the normal glucose condition (Figure 2C). These results indicate that metformin rectifies high glucose–induced VSMC migration enhancement via the HMGB1-autophagy related pathway.

### Metformin inhibited HMGB1 expression by increasing miR-142-3p expression in VSMC

**Figure 3:**
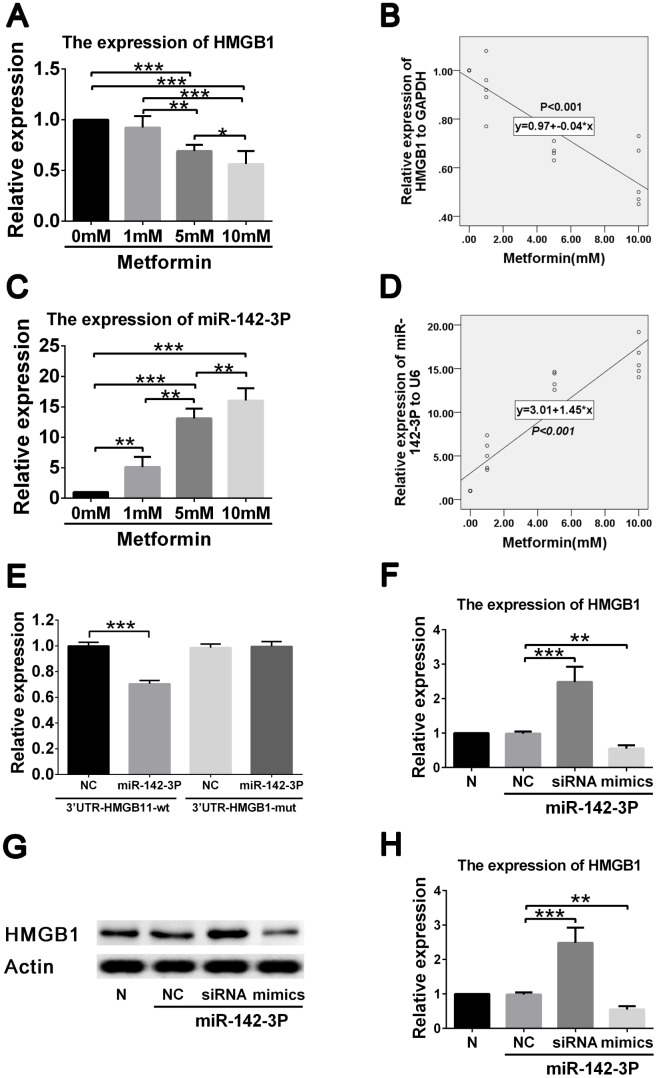
**Metformin exerts effects on HMGB1 via affecting miR-142-3p in vascular smooth muscle cells (VSMCs).** (**A**) Decreased HMGB1 gene expression in a metformin dose-dependent manner by quantitative real-time PCR. (**B**) Correlation analysis revealed that negative correlation was found between metformin concentration and HMGB1 gene expression. (**C**) Increased miR-142-3p expression in a metformin dose-dependent manner by quantitative real-time PCR. (**D**) Correlation analysis revealed that positive correlation was found between metformin concentration and miR-142-3p gene expression. (**E**) Decreased HMGB1 level was found in HMGB1-WT-3′UTR + miR-142-3p transfected VSMCs compared to HMGB1-WT-3′UTR + control vector transfected VSMCs, whereas HMGB1 level was similar between HMGB1-MUT-3′UTR + miR-142-3p transfected VSMCs and HMGB1- MUT -3′UTR + control vector transfected VSMCs. (**F**) miR-142-3p overexpression and inhibition by mimics and siRNA resulted in decreased and increased HMGB1 gene expression in VSMCs, respectively. (**G**) miR-142-3p overexpression and inhibition by mimics and siRNA resulted in decreased and increased HMGB1 protein expression in VSMCs, respectively. (**H**) Relative quantification of HMGB1 level. **p < 0.01 and ***p < 0.001 for between group comparison.

To further clarify the regulatory network involved in the effects of metformin on high glucose–induced VSMC proliferation and migration, we performed gene expression analysis and correlation analysis between HMGB1, and miR-142-3p expression and metformin concentration. A negative correlation was found between HMGB1 gene level and metformin concentration (Figure 3A, 3B), and a positive correlation was found between miR-142-3p level and metformin concentration (Figure 3C, 3D).

To confirm that HMGB1 is regulated by miR-142-3p, reporter assays were performed. Results showed that miR-142-3p mimics significantly reduced the expression of HMGB1 (Figure 3E). miR-142-3p was also shown to inhibit HMGB1 expression by directly binding to its 3′-UTR segment. To confirm the ability of miR-142-3p to suppress HMGB1 expression, VSMCs were transfected with miR-142-3p mimics and inhibitor (Figure 3F). The group containing miR-142-3p mimics displayed a robustly decreased HMGB1 protein level, whereas VSMCs in the group with miR-142-3p inhibitor demonstrated an increased HMGB1 protein level (Figure 3G, 3H). These results indicate that metformin decreases HMGB1 expression by promoting miR-142-3p in VSMCs.

### miR-142-3p overexpression by mimic and inhibition by siRNA result in inhibition and promotion, respectively of high glucose–induced VSMCs migration via the Akt/PI3K/autophagy related pathway

**Figure 4:**
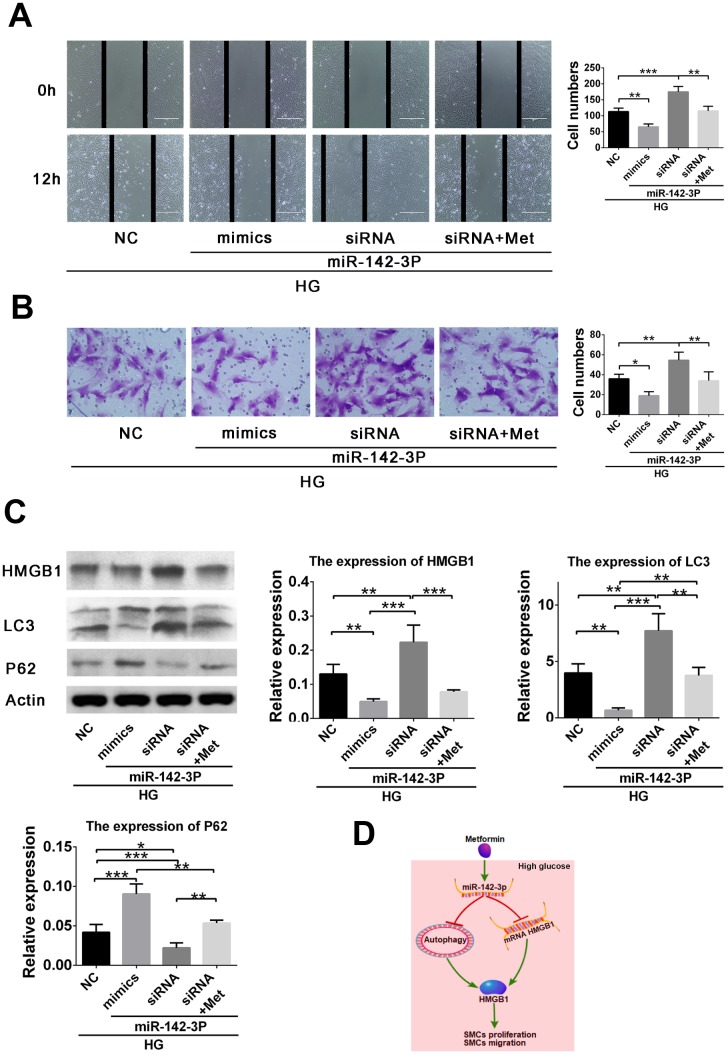
**miR-142-3p overexpression by mimic and miR-142-3p inhibition by siRNA resulted in inhibition and promotion, respectively, of high glucose–induced vascular smooth muscle cell (VSMC) migration enhancement via the HMGB1-autophagy related pathway, whereas metformin abolished the effects of miR-142-3p siRNA.** (**A**) Scratch assay for the effects of miR-142-3p overexpression and inhibition. (**B**) Transwell assay for the effects of miR-142-3p overexpression and inhibition. (**C**) miR-142-3p overexpression by mimic results in decreased HMGB1 and LC3II and elevated p62 level in high glucose–induced VSMC migration enhancement, whereas miR-142-3p siRNA caused the opposite effects and metformin abolished the effects of miR-142-3p siRNA. (**D**) Schematic of the role of metformin in the regulation of VSMC proliferation and migration. *p < 0.05, **p < 0.01 and ***p < 0.001 for between-group comparison.

To further verify the role of miR-142-3p in high glucose–induced VSMC migration, miR-142-3p overexpression and inhibition were induced by mimic and siRNA transfection, respectively. We found that miR-142-3p overexpression by mimic and inhibition by siRNA resulted in inhibition and promotion, respectively, of high glucose–induced VSMC migration via the HMGB1-autophagy related pathway. However, metformin abolished the effects of miR-142-3p siRNA (Figure 4A, 4B). Further analysis revealed that miR-142-3p overexpression by mimic resulted in inhibition of HMGB1 and LC3II levels and elevated p62 level in high glucose–induced VSMC migration enhancement. However, miR-142-3p siRNA generated the reverse effects, and metformin abolished the effects of miR-142-3p siRNA (Figure 4C). These results confirm the effects of miR-142-3p on the migration behavior of VSMCs in the high-glucose condition.

## DISCUSSION

Abnormal proliferation and migration of VSMC, which is enhanced by inflammation in hyperglycemic states, contribute to the formation of atherosclerotic lesions [24]. According to previous studies, metformin attenuates early-stage atherosclerosis in mildly hyperglycemic Oikawa-Nagao mice [17]. This may be due to the inhibition of the inflammatory response and vascular calcification in VSMC by metformin [24, 25]. Metformin also inhibits proliferation of tumor and endothelial cells [26, 27]. In our study, metformin decreased VSMC proliferation in the high-glucose condition. In addition, VSMC migration was also inhibited by metformin. Finally, increased miR-142-3p expression was shown to inhibit VSMC proliferation and migration via the HMGB1-autophagy related pathway (Figure 4D).

HMGB1 is an inflammatory factor that increase VSMC proliferation and high glucose–induced calcification in VSMC and subsequent vascular inflammation and atherosclerosis [4, 28, 29]. In addition, HMGB1 has been identified as an autophagy sensor in oxidative stress [30], and autophagy may regulate the expression and release of HMGB1 [31]. However, increasing evidences indicates that HMGB1 is downregulated by metformin [23]. In our previous studies, we found that metformin induced autophagy by activating AMPK, resulting in decreased proliferation and migration of endothelial progenitor cells [32, 33]. In the present study, we also found that metformin inhibits the proliferation and migration of VSMC induced by elevated glucose. This might be related to the autophagy pathway promoted by metformin.

It has been well demonstrated that many microRNAs affect atherosclerosis by inhibiting cardiovascular inflammation [13, 34]. MiR-142-3p, one of the novel inflammation-related miRNAs [35], is significantly upregulated by metformin [36]. It may inhibit cell proliferation and migration via the WNT signaling pathway and autophagy [37–39]. In addition, miR-142-3p may target HMGB1 in tumor cells [14, 40]. Consistently, we found that miR-142-3p inhibited migration enhancement in high glucose–stimulated VSMC. These findings validate that miR-142-3p is involved in the regulation of abnormal VSMC proliferation and migration, which contribute to ASO and in-stent restenosis. However, these findings contradict some previous studies. For example, Bao et al found that miR-142-3p promoted endothelial cell proliferation via Bcl-2–associated transcription factor 1 [41]. In addition, Wu et al found that miR-142-3p promoted the neuronal cell cycle and inhibited apoptosis after peripheral nerve injury [42]. Thus, further investigation, especially the appropriate animal model studies, of microRNAs involved in the regulation of cell growth should be performed to clarify these issues. In conclusion, we demonstrated that metformin inhibits high glucose–induced VSMC hyper-proliferation and migration enhancement by promoting miR-142-3p–mediated inhibition of HMGB1 expression via the autophagy pathway.

## MATERIALS AND METHODS

### VSMC isolation and characterization

All research involving experimental animals was approved by the Institutional Review Board of the Drum Tower Hospital Affiliated to Medical School of Nanjing University, Nanjing, China, and adhere to the international experiment guideline. VSMCs, obtained from the aortic artery, were cultured with DMEM containing 20% FBS at 37°C. Immunofluorescence with α-actin was carried out to identify the cells [43].

### Cell treatment

VSMCs were grown to 70%-80% cell confluence and exposed to normal glucose (5.6 + 19.4 mmol/L mannitol) or High Glucose (25 mmol/L) for 24 hours. Metformin was added to the high glucose–treated cells. In the cell proliferation assay, metformin was used at a serial concentration of 1, 5, and 10 mM. The concentration of metformin in the cell migration assay was 5 mM.

### CCK-8 assay for cell growth

Trypsinized VSMCs (2 × 10^5^ cells) were resuspended in complete VSMC medium and seeded in a 12-well plate and incubated with 0, 1, 5 and 10 mM of metformin in the previously mentioned normal and high-glucose conditions under 37°C, 5% CO_2_. One day later, cell proliferation was evaluated by CCK-8 assay following the manufacturer’s instructions. At least triplicate repeats were completed in all the experiments.

### Wound healing assay

VSMCs were allowed to be grown to 80%-90% cell confluence and treated with normal glucose or high glucose in the absence or presence of metformin. A scratch was made by a 200 μL pipette tip at 0 h and incubated at 37°C, 5% CO_2_. Pictures were taken using microscopy at 0 and 24 h for migration evaluation.

### Transwell assay

A modified transwell assay in 24-well plates was used for cell migration evaluation (BD Biosciences, San Jose, CA, USA). After cell transfection for 24 h, cells were suspended with serum-free DMEM at a concentration of 3 × 10^5^ cells and added to the upper chamber, while the lower chamber was filled with DMEM supplemented with 20% FBS as a chemoattractant. Then the transwell plate was incubated at 37°C for 24 h, and a cotton swab was used to remove upper chamber residue cells. The transwell membrane close to the lower chamber was cut by scissors, stained with crystal violet and photographed by a microscope. The cells were then counted according to the pictures.

### Cell transfection

miR-142-3p mimic, siRNA, and their negative controls were purchased from Thermo. miR-142-3p mimic (50 nM), siRNA (150 nM), and their negative controls were transfected, into VSMCs at 80% confluence using Lipofectamine 3000 (Invitrogen, Carlsbad, CA, USA) according to the manufacturer’s instructions. After 48 h of transfection, cells were harvested for subsequent experiments, and the expression of microRNA was confirmed by real-time reverse transcriptase quantitative polymerase chain reaction (RT-qPCR), described later.

### Luciferase assay

Luciferase reporter assay was performed, as previously described [44], to explore the potential regulation mechanisms of miR-142-3p. For the measurement of luciferase activity, cells were cotransfected in 24-well plates with 100 ng of luciferase plasmid and 50 ng of Renilla plasmid (Ambion) as a control, as well as with 400 ng of miR-142-3p mimics or negative control microRNA. The luciferase and Renilla plasmid activities were measured 48 h later using the Dual Luciferase Reporter 1000 Assay System (Promega, Madison, WI, USA).

### RT-qPCR

RT-qPCR was performed as previously reported [44, 45]. Briefly, total RNA was isolated from the VSMCs treated with the series of metformin concentration. Real-time PCR detection was performed when generation of cDNA for RT-qPCR was done. GAPDH expression and U6 expression were used as controls for HMGB1 and microRNA gene, respectively. Relative microRNA expression was normalized to the reference microRNA expression using the ΔΔCt method. All primer sequences arelisted in Table 1.

**Table 1:** Primer sequence.

**Gene name**	**Forward**	**Reverse**
HMGB1	5′-AGCAATCTGAACGTCTGTCC-3′	5′-GTTCTTGTGATAGCCTTCGC-3′
GAPDH	5′-GCGCTGAGTACGTCG-3′	5′-CAGTTGGTGGTGCAG-3′
MiR-142-3p	TGTAGTGTTTCCTACTTTATGGA	
U6	CTCGCTTCGGCAGCACA	AACGCTTCACGAATTTGCGT

### Western blot analysis

Cellar protein was extracted from VSMCs (1 × 10^5^ cells), as previously reported [44]. Briefly, the proteins were separated and then transferred. After blocking, membranes were incubated with HMGB1(Cell Signaling Technology [CST], Danvers, MA, USA), LC3II/I(CST), p62(CST), and Actin (Sigma, St. Louis, MO, USA). Appropriate secondary antibodies were used. The protein bands were detected using an Infrared Imaging System (LI-COR).

### Statistical analysis

All statistical analyses were performed using SPSS v21. Data are presented as mean ± SD. Student’s t test or one-way ANOVA was used to examine the differences between the groups. Correlations between HMGB-1 and miR-142-3p levels and metformin concentration were analyzed using the Pearson correlation method. *P* < 0.05 was considered as statistically significant.
